# Supplementary material for: Identification of photoperiod-induced specific miRNAs in the adrenal glands of Sunite sheep (Ovis aries)
Source: Front Vet Sci. 2022 Jul 22;9:888207. doi: 10.3389/fvets.2022.888207 (PMC9354845; doi:10.3389/fvets.2022.888207)
Supplement: Supplementary file 1 [file Data_Sheet_1.ZIP › Supplementary file/miRDeep2 file.zip!/miRDeep2 file/Know_Structure/SP42A1/oar-mir-136.pdf]

miRBase precursor : oar-mir-136
Total read count : 5869
oar-miR-136 read count : 1302
remaining reads : 4567

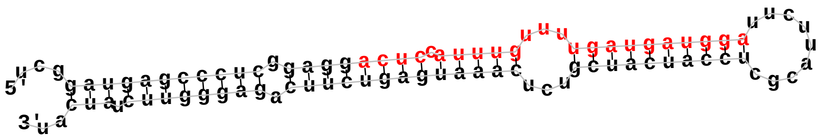

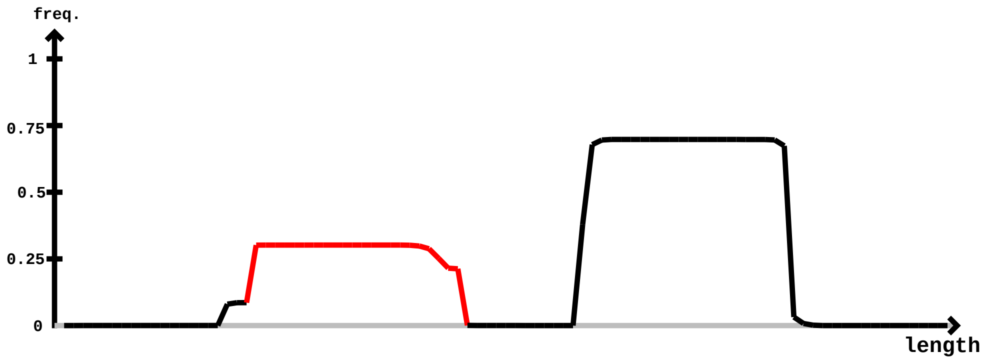


```
                                    oar-miR-136
5'- ucggaugagcccucggaggacuccauuuguuuugaugauggauucuuacgcuccaucaucgucucaaaugagucuucagaggguucuaucau   -3'    exp
    ...(((((((((((.((((((((.(((((...((((((((((.........))))))))))...))))))))))))).)))))))).)))..     reads     mm     sample
    .cggaugagcccucggagg.........................................................................     1         0      seq
    ................aggacuccauuuguuuuga.........................................................     4         0      seq
    ................aggacuccauuuguuuugau........................................................     18        0      seq
    ................aggacuccauuuguuuugaug.......................................................     58        0      seq
    ................aggacuccauuuguuuugauga......................................................     206       0      seq
    ................aggacuccauuuguuuugaugau.....................................................     184       0      seq
    .................ggacuccauuuguuuugaug.......................................................     2         0      seq
    .................ggacuccauuuguuuugauga......................................................     4         0      seq
    .................ggacuccauuuguuuugaugau.....................................................     26        0      seq
    ..................gacuccauuuguuuugaugaug....................................................     1         0      seq
    ...................acuccauuuguuuugaugau.....................................................     7         0      seq
    ...................acuccauuuguuuugaugaug....................................................     13        0      seq
    ...................acuccauuuguuuugaugaugg...................................................     1244      0      seq
    ...................acuccauuuguuuugaugaugga..................................................     3         0      seq
    ....................cuccauuuguuuugaugaugg...................................................     1         0      seq
    .........................uuuguuuugaugauggauucuu.............................................     1         0      seq
    .....................................................caucaucgucucaaaug......................     2         0      seq
    .....................................................caucaucgucucaaaugagu...................     8         0      seq
    .....................................................caucaucgucucaaaugaguc..................     105       0      seq
    .....................................................caucaucgucucaaaugagucu.................     2098      0      seq
    .....................................................caucaucgucucaaaugagucuu................     13        0      seq
    ......................................................aucaucgucucaaaugagu...................     1         0      seq
    ......................................................aucaucgucucaaaugaguc..................     24        0      seq
    ......................................................aucaucgucucaaaugagucu.................     1637      0      seq
    ......................................................aGcaucgucucaaaugagucu.................     3         1      seq
    ......................................................aucaucgucucaaaugagucuu................     90        0      seq
    ......................................................aucaucgucucaaaugagucuuc...............     1         0      seq
    .......................................................ucaucgucucaaaugaguc..................     1         0      seq
    .......................................................ucauUgucucaaaugagucu.................     1         1      seq
    .......................................................ucaucgucucaaaugagucu.................     29        0      seq
    .......................................................ucaucgucucaaaugagucuu................     36        0      seq
    .......................................................ucaucgucucaaaugagucuuc...............     29        0      seq
    .......................................................ucaucgucucaaaugagucuuca..............     5         0      seq
    ........................................................caucgucucaaaugagucuu................     2         0      seq
    ........................................................caucgucucaaaugagucuuc...............     7         0      seq
```

oar-miR-136

```
ucggaugagcccucggaggacuccauuuguuuugaugauggauucuuacgcuccaucaucgucucaaaugagucuucagaggguucuaucau
........................................................caucgucucaaaugagucuuca..............    4    0    seq
```
